# Association between psychopharmacotherapy and postpartum hemorrhage

**DOI:** 10.1016/j.xagr.2024.100402

**Published:** 2024-10-09

**Authors:** Frank I. Jackson, Insaf Kouba, Natalie Meirowitz, Nathan A. Keller, Luis A. Bracero, Matthew J. Blitz

**Affiliations:** 1 Northwell, New Hyde Park, NY (Jackson, Kouba, Meirowitz, Keller, Bracero, and Blitz); 2 Department of Obstetrics and Gynecology, South Shore University Hospital, Bay Shore, NY (Jackson, Kouba, Keller, Bracero, and Blitz); 3 Zucker School of Medicine, Hempstead, NY (Jackson, Kouba, Meirowitz, Keller, Bracero, and Blitz); 4 Department of Obstetrics and Gynecology, Long Island Jewish Medical Center, New Hyde Park, NY (Meirowitz); 5 Institute of Health Systems Science, Feinstein Institutes for Medical Research, Northwell Health, Manhasset, NY (Blitz)

**Keywords:** benzodiazepine, bleeding risk assessment, dopamine-norepinephrine reuptake inhibitors, perinatal mood disorder, mental health condition, selective serotonin reuptake inhibitors, serotonin-norepinephrine reuptake inhibitors

## Abstract

**Background:**

Prior studies evaluating the relationship between psychopharmacotherapy (PPT), and postpartum hemorrhage (PPH) have yielded inconsistent findings. Clarifying this potential relationship is important for effective counseling and risk stratification.

**Objectives:**

Our primary objective was to evaluate the association between prenatal exposure to PPT (any drug class) and the occurrence of PPH requiring transfusion of packed red blood cells (PPH+pRBC) after systematically adjusting for known hemorrhage risk factors at the time of admission for delivery. Secondary objectives were to evaluate the association between individual PPT drug classes and PPH+pRBC, and the association between treatment intensity of mental health condition and PPH+pRBC. Finally, we evaluated the association between PPT and a broader definition of PPH that included deliveries requiring multiple uterotonic drugs.

**Study design:**

This is a retrospective cross-sectional study of all pregnancies delivered at 23 weeks of gestational age or greater at seven hospitals within a large academic health system in New York between January 2019 and December 2022. There were no exclusion criteria, as postpartum hemorrhage risk assessment is necessary for all patients admitted for delivery. We assessed exposure to prenatal PPT, including selective serotonin reuptake inhibitors (SSRIs: escitalopram, fluoxetine, sertraline), serotonin-norepinephrine reuptake inhibitors (SNRIs: duloxetine, venlafaxine), dopamine-norepinephrine reuptake inhibitors (DNRIs: buproprion), benzodiazepines (alprazolam, diazepam, lorazepam), and others (buspirone, trazodone, zolpidem). Multivariable logistic regression was performed to evaluate the relationship between PPT and PPH+pRBC, while systematically adjusting for known hemorrhage risk factors at the time of hospital admission. Similar regression analyses were performed to address the secondary objectives.

**Results:**

A total of 107,425 deliveries were included. Non-Hispanic White patients constituted the largest race and ethnicity group (43.4%), followed by Hispanic patients (18.7%), Asian or Pacific Islander patients (13.2%), and non-Hispanic Black patients (12.3%). Prenatal exposure to PPT occurred in 3.6% of pregnancies (*n*=3,834). The overall rate of PPH+pRBC was 2.9% (*n*=3,162). PPH+pRBC occurred more frequently in pregnancies exposed to PPT than in pregnancies which were not exposed (5.5% vs. 2.8%, respectively; aOR 2.10, 95% CI: 1.79–2.44). SSRIs and benzodiazepine monotherapy were each associated with higher odds of PPH+pRBC than nonexposure. Compared to patients without a mental health condition, monotherapy was associated with nearly 2-fold increased odds and combination PPT was associated with nearly 4-fold greater odds of PPH+pRBC after adjustment for confounding variables (monotherapy: aOR 1.94, 95% CI: 1.64–2.28; combination PPT: aOR 3.96, 95% CI: 2.61–5.79). Patients with untreated mental health conditions (no PTT) had no increased odds of PPH+pRBC compared to those without mental health conditions. Finally, after adjusting for covariates, a positive association was found between PPT and PPH requiring pRBC transfusion and/or the use of two additional uterotonic agents beyond routine postpartum oxytocin (aOR 1.53, 95% CI: 1.35–1.73).

**Conclusions:**

Prenatal PPT exposure is associated with increased odds of clinically significant PPH+pRBC after adjusting for other hemorrhage risk factors. Combination PPT was associated with greater odds of PPH+pRBC than monotherapy.

**AJOG Global Reports at a Glance**

**Why was this study conducted?**

The risk of postpartum hemorrhage (PPH) in patients exposed to psychopharmacotherapy (PPT) is unclear in the literature. Clarifying this risk is important for patient counseling and risk stratification.

**Key findings**

In this retrospective cross-sectional study of 107,589 deliveries, exposure to PPT was associated with an increased odds of PPH requiring transfusion of packed red blood cells (PPH+pRBC) compared to no exposure after adjusting for known hemorrhage risk factors. Combination PPT was associated with greater odds of PPH+pRBC than monotherapy.

**What does this study add to what is already known?**

Prenatal PPT is associated with clinically significant PPH and should be considered for inclusion in risk assessment scoring tools.

## Introduction

Mental health conditions are common in pregnancy, and their prevalence is increasing nationally.^1^ In recent decades, there has also been a concomitant rise in the use of psychopharmacotherapy (PPT), the use of pharmacological agents to treat mood and anxiety disorders.^2^ Several classes of drugs are encountered during pregnancy, including selective serotonin reuptake inhibitors (SSRIs), serotonin-norepinephrine reuptake inhibitors (SNRIs), dopamine-norepinephrine reuptake inhibitors (DNRIs), benzodiazepines, and others. There is extensive, albeit imperfect, literature^3^, ^4^, ^5^, ^6^, ^7^, ^8^ regarding the potential effect of prenatal exposure to these medications and risk of teratogenesis, childhood neurodevelopmental outcomes, and other pregnancy complications, such as postpartum hemorrhage (PPH), the leading cause of maternal mortality worldwide.^9^

In nonpregnant patients, spontaneous and perioperative bleeding complications are associated with use of SSRIs and SNRIs in some, but not all, observational studies.^10^, ^11^, ^12^, ^13^ In pregnant patients, prior studies evaluating the relationship between PPT and PPH have similarly yielded inconsistent findings and the magnitude of risk, if any, remains unclear.^14^, ^15^, ^16^, ^17^, ^18^, ^19^, ^20^ Serotonin, or 5-hydroxytryptamine (5-HT), plays several roles throughout the body. In the central nervous system, it helps to regulate mood and emotions. In the vascular system, injury causes platelets to release serotonin, which triggers vasoconstriction and platelet aggregation.^21^ SSRIs and SNRIs increase serotonin levels in the brain by blocking its reabsorption into neurons, but these medications also inhibit the uptake of serotonin into platelets which may impair hemostatic function.^22^ Inconsistent findings in prior studies may be attributable to the heterogenous patient populations being studied, how the bleeding outcomes are defined, and how many patients are needed to detect rare events. Studies that include low-risk bleeding events are more likely to find positive associations. Better elucidating the risk of clinically relevant, life-threatening adverse events should be a priority.

Our primary objective was to evaluate the association between prenatal exposure to PPT (any drug class) and the occurrence of PPH requiring transfusion of packed red blood cells (PPH+pRBC) after systematically adjusting for known hemorrhage risk factors at the time of admission for delivery. Secondary objectives were to evaluate the association between individual PPT drug classes and PPH+pRBC, and the association between treatment intensity of mental health condition and PPH+pRBC. Finally, we evaluated the association between PPT and a broader definition of PPH that included deliveries requiring multiple uterotonic drugs.

## Materials and methods

This was a retrospective cross-sectional study of all pregnant patients who delivered between January 2019 and December 2022 at 7 hospitals within a large academic health system in New York, comprised of both community and tertiary care facilities. Patients with deliveries at 23 weeks of gestational age or greater were included. There were no exclusion criteria, as postpartum hemorrhage risk assessment is needed for all patients admitted for delivery. The local institutional review board approved this study as minimal-risk research and waived the requirement for informed consent. The study followed the Strengthening the Reporting of Observational Studies in Epidemiology (STROBE) reporting guideline.

### Data sources

Clinical and sociodemographic data were obtained from the inpatient electronic medical record system (Sunrise Clinical Manager, Allscripts Corp., Chicago, IL). Baseline characteristics included maternal age, obesity (yes/no), public health insurance (yes/no), and self-identified race and ethnicity which were selected from prespecified categories at the time of hospital admission. Advanced maternal age (AMA) was defined as ≥35 years at delivery. Obesity was defined as ≥30 kg/m^2^. Medication exposures were determined by their presence or absence in the medication reconciliation document completed during hospital admission.

### Exposure

The primary exposure of interest (primary independent variable) was prenatal exposure to PPT (yes/no). The twelve most commonly used PPT drugs were selected for evaluation from the following drug classes: SSRIs (escitalopram, fluoxetine, sertraline), SNRIs (duloxetine, venlafaxine), DNRIs (buproprion), benzodiazepines (alprazolam, diazepam, lorazepam), and other (buspirone, trazodone, zolpidem).^2^

### Potential confounders or effect-modifiers

The other independent variables were selected from admission risk factors in the Association of Women's Health, Obstetric and Neonatal Nurses (AWHONN) PPH risk assessment tool^23^ and included the following binary variables: induction of labor, multiple gestation, >4 previous vaginal births, prior cesarean or uterine incision, large uterine fibroids, fetal demise, polyhydramnios, placental abruption, placenta accreta, placenta previa, known coagulopathy, anemia (hematocrit <30%), and thrombocytopenia (platelets <100,000/mm^3^). AWHONN does not provide an explicit definition for large fibroid; for this study, fibroids >5 cm in diameter, based on either clinical documentation or ultrasound report, were included given recent evidence of increased PPH risk beyond this size threshold.^24^ Personal and family history of PPH were not evaluated due to inconsistent documentation and potential for recall bias. Chorioamnionitis was not included in the primary analysis because it is typically characterized as an intrapartum rather than admission diagnosis.

### Outcome

The primary outcome (dependent variable) was PPH+pRBC, which was determined by the presence of an ICD-10 diagnosis code for PPH and a procedure code for blood transfusion. Blood transfusions are generally recommended for symptomatic postpartum patients with hemoglobin levels below 8 mg/dL or acute ongoing obstetrical blood loss requiring stabilization. A secondary outcome was PPH requiring intervention (PPH+intervention); this definition included PPH cases with pRBC transfusion and/or the use of two uterotonic agents in addition to routine postpartum oxytocin. Some providers may administer one additional uterotonic prophylactically, but the authors believe it less likely for two medications to be given in this manner.

### Statistical analysis

Descriptive statistics including frequencies and percentages were calculated to summarize the characteristics of the study population. Odds ratios (ORs) and 95% confidence intervals (CIs) were estimated to quantify the strength and direction of associations. Statistical significance was defined as a *P*-value <.05. All analyses were performed in R version 4.3.1.

For our primary objective, first, an unadjusted logistic regression model evaluated the association between PPT and PPH+pRBC without considering any other independent predictors. Second, a multivariate logistic regression model evaluated the relationship between PPT and PPH+pRBC, adjusting for each of the individual AWHONN risk factors described previously and additional clinical and sociodemographic variables, including AMA, obesity, public health insurance, and race and ethnicity group.

For our secondary objectives, further analyses were performed. First, a multivariate logistic regression model assessed monotherapy with individual PPT drug classes (SSRIs, SNRIs, DNRIs, benzodiazepines) and their association with PPH+pRBC. Second, multivariate logistic regression was used to evaluate the relationship between treatment intensity of mental health condition (ordinal predictor variable: no PPT, monotherapy, combination PPT) and PPH+pRBC. Finally, multivariate logistic regression was used to evaluate the relationship between PPT and PPH+intervention, to confirm that our findings persist with a broader PPH definition.

In the interest of transparency, additional supplemental analyses were performed to clarify the role of variables excluded from our primary and secondary objectives (i.e., intrapartum factors and other variables not included in the AWHONN risk assessment tool). The final model from our primary objective was modified to adjust for chorioamnionitis, mode of delivery, macrosomia, hypertensive disorders of pregnancy, and excessive gestational weight gain; this “expanded model” should be interpreted cautiously because some of the additional variables are mediators between exposure and outcome. We also performed additional analysis on the subset of patients without any PPT exposure, and evaluated whether mental health condition, in the absence of PPT exposure, was associated with PPH+pRBC.

## Results

### Descriptive data

**Figure 1:**
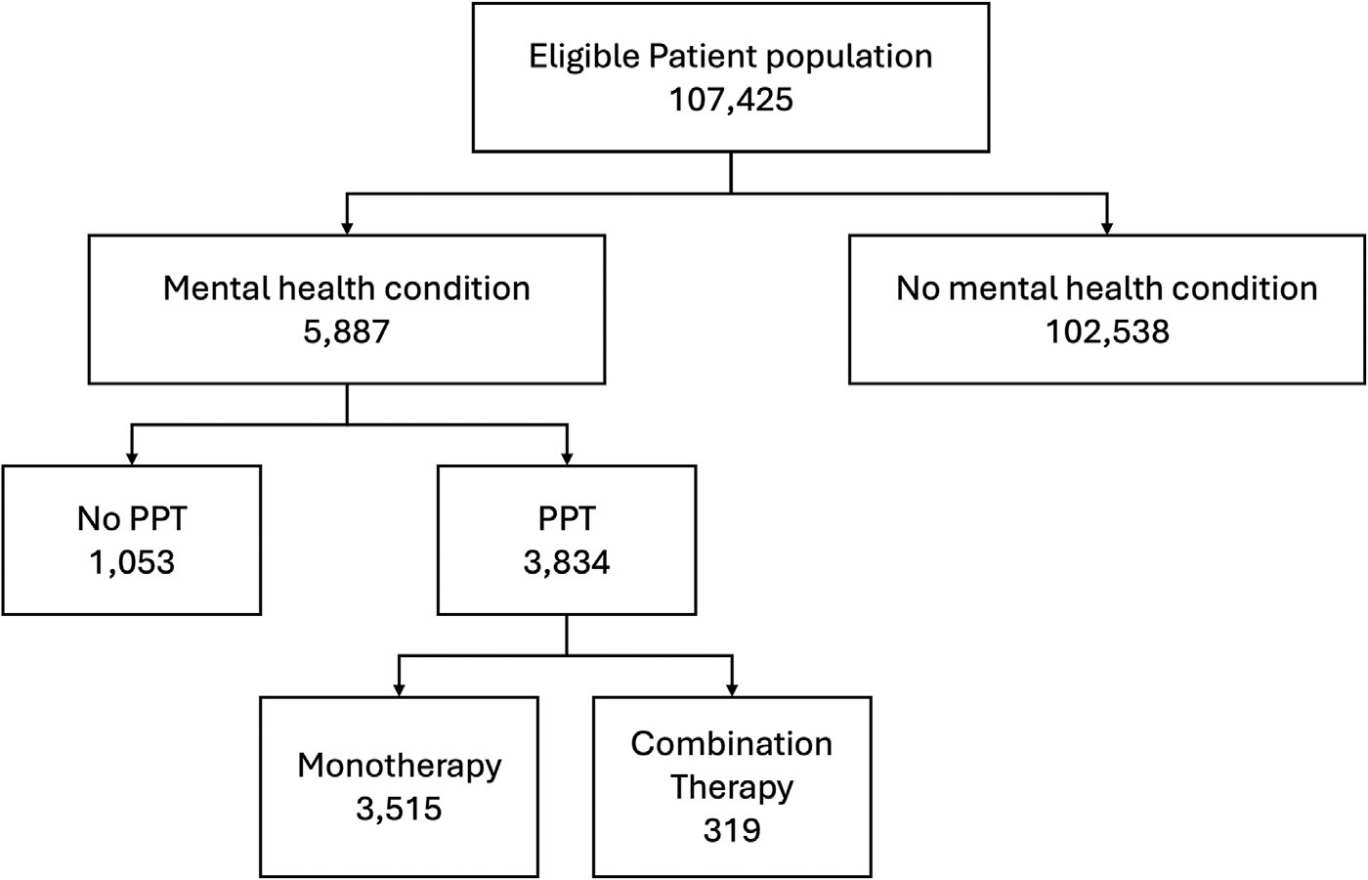
Patient flowchart

**Figure 2:**
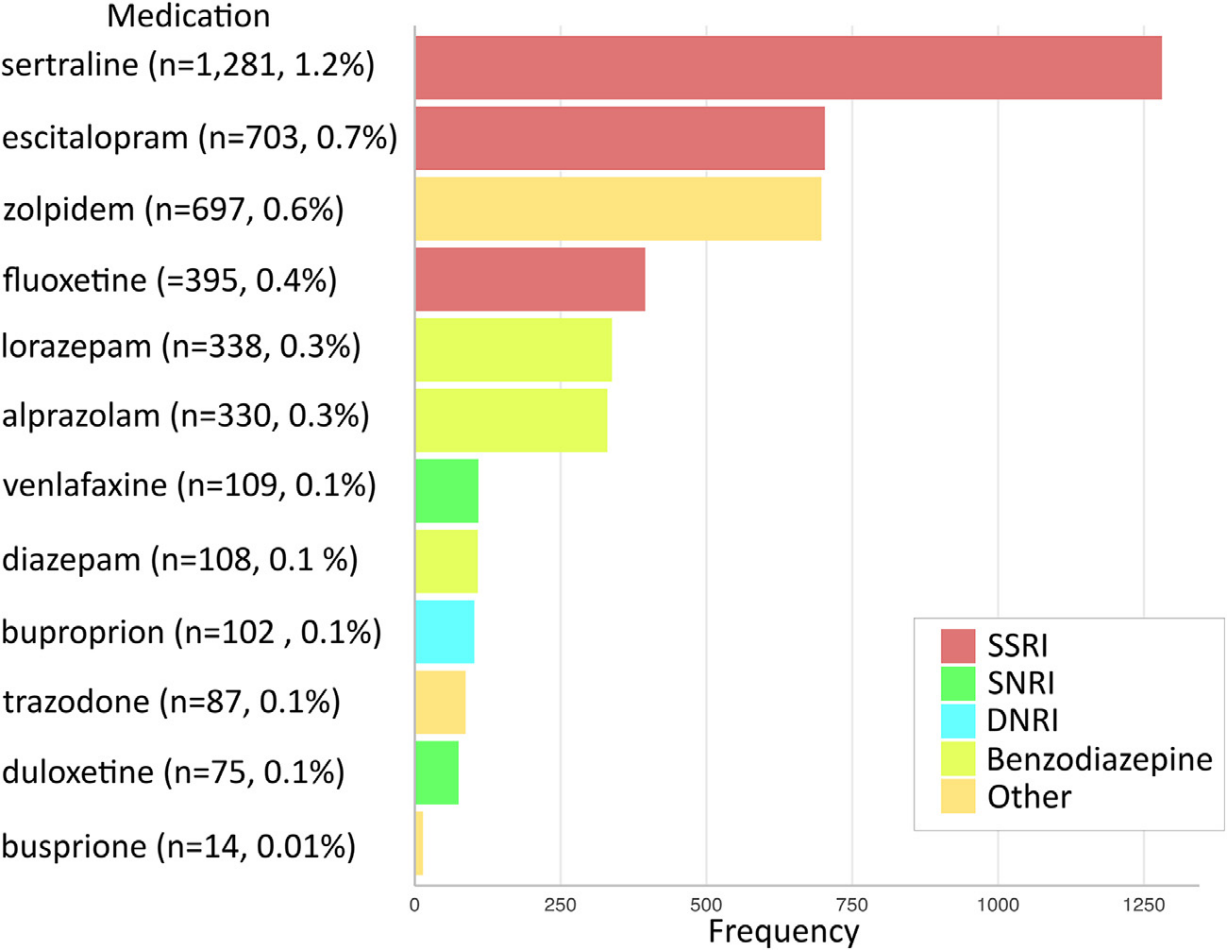
Psychopharmacotherapy medications used by study population

A total of 107,425 deliveries were included for analysis. Patient flowchart is shown in Figure 1. Non-Hispanic White patients constituted the largest race and ethnicity group (43.4%), followed by Hispanic patients (18.7%), Asian or Pacific Islander patients (13.2%), and non-Hispanic Black patients (12.3%). Prenatal exposure to PPT occurred in 3.6% of pregnancies (*n*=3,834). Mental health condition in the absence of PPT exposure occurred in 1.0% of pregnancies (*n*=1,029). The overall rate of PPH+pRBC was 2.9% (*n*=3,162) and the rate of PPH+intervention was 6.1% (*n*=6,525). Baseline characteristics of the study population are summarized in Table 1. Psychopharmacotherapy medications used by the study population are shown in Figure 2.

Jackson. Association between psychopharmacotherapy and postpartum hemorrhage. Am J Obstet Gynecol MFM 2024.

**Table 1:** Baseline characteristics

Characteristic	Did not receive PPT (*n*=103,591)	Received PPT (*n*=3,834)	*P*-value
Age, years	31.6 (±5.4)	32.9 (±5.3)	<.001
>35	31,577 (30.5)	1,488 (38.3)	<.001
Race and ethnicity			
American Indian or Alaska Native	631 (0.6)	8 (0.2)	<.001
Asian or Pacific Islander	13,984 (13.5)	185 (4.8)	
Hispanic	19,479 (18.8)	375 (9.8)	
Non-Hispanic Black	12,918 (12.47)	250 (6.5)	
Non-Hispanic White	43,932 (42.4)	2,667 (69.6)	
Other or multiracial	8,952 (8.6)	225 (5.9)	
Declined or unknown	3,425 (3.3)	124 (3.2)	
Public insurance	34,494 (33.3)	964 (25.1)	<.001
Obesity	49164 (47.5)	1,883 (49.1)	.046
AWHONN PPH risk factor			
Induction of labor	35,868 (34.6)	1,322 (34.5)	.87
Multiple gestation	1,887 (1.8)	79 (2)	.31
>4 Previous vaginal births	3,110 (3.0)	141 (3.7)	.02
Prior uterine surgery	16,387 (15.8)	643 (16.8)	.12
Large fibroid (>5 cm)	1,303 (1.3)	43 (1.1)	.50
Chorioamnionitis	1,701 (1.6)	35 (0.9)	.006
Polyhydramnios	1,394 (1.4)	102 (2.7)	<.001
Fetal demise	282 (0.3)	50 (1.3)	<.001
Anticoagulation	606 (0.6)	50 (1.3)	<.001
Placental abruption, accreta, previa	2,014 (1.9)	108 (2.8)	<.001
Anemia	5,988 (5.8)	286 (7.5)	<.001
Hematocrit, %	35.8 (±3.6)	33.2 (±3.6)	<.001
Thrombocytopenia	481 (0.5)	21 (0.6)	.53
AWHONN PPH risk category			
Low	45,441 (43.9)	1,549 (40.4)	<.001
Moderate	46,972 (45.3)	1,778 (46.4)	
High	11,178 (10.8)	507 (13.2)	

PPT, psychopharmacotherapy; AWHONN, Association of Women's Health, Obstetric and Neonatal Nurses; PPH, postpartum hemorrhage.

Data are number (percent) or mean (±standard deviation).

Jackson. Association between psychopharmacotherapy and postpartum hemorrhage. Am J Obstet Gynecol MFM 2024.

### Primary analysis

**Figure 3:**
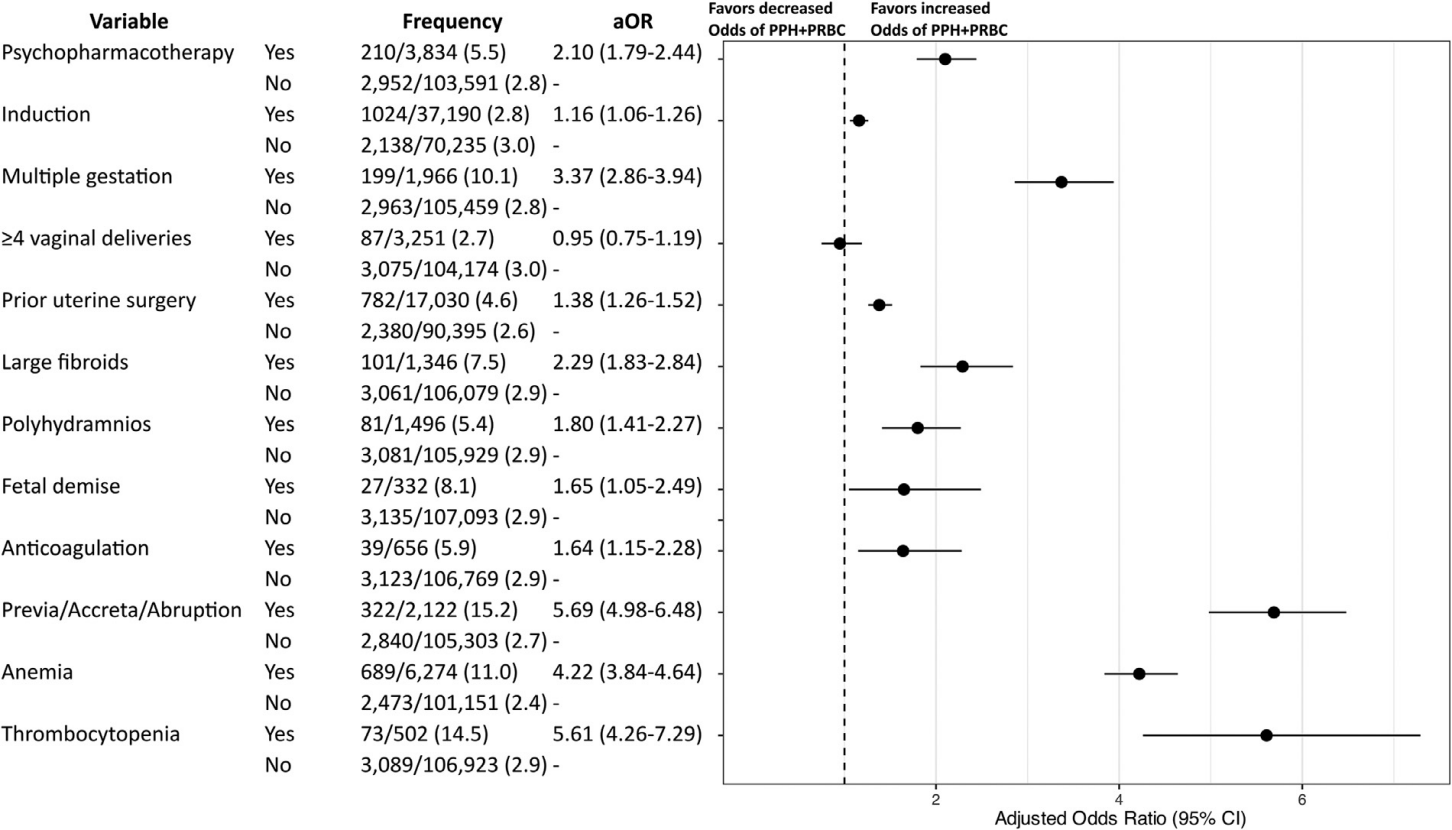
Regression model evaluating association between psychopharmacotherapy (PPT) and postpartum hemorrhage requiring packed red blood cells (PPH+pRBC), adjusting for PPH risk factors at admission and other clinical and sociodemographic variables *Variables shown in the forest plot are those included in the Association of Women's Health, Obstetric and Neonatal Nurses (AWHONN) postpartum hemorrhage risk assessment tool. *The following variables were adjusted for in the model, but the results are not shown: advanced maternal age, obesity, public health insurance, and race and ethnicity group.

Among pregnancies exposed to PPT, 5.5% (*n*=210/3,834) were complicated by PPH+pRBC, compared to 2.8% (*n*=2,952/103,591) among nonexposed pregnancies. Before adjustment for confounding factors, prenatal PPT exposure approximately doubled the odds of PPH+pRBC compared to patients without exposure (OR 2.06, 95% CI: 1.76–2.40). After adjustment for individual PPH risk factors, the results were similar (aOR 2.10, 95% CI: 1.79–2.44; Figure 3).

Jackson. Association between psychopharmacotherapy and postpartum hemorrhage. Am J Obstet Gynecol MFM 2024.

### Secondary analysis

**Figure 4:**
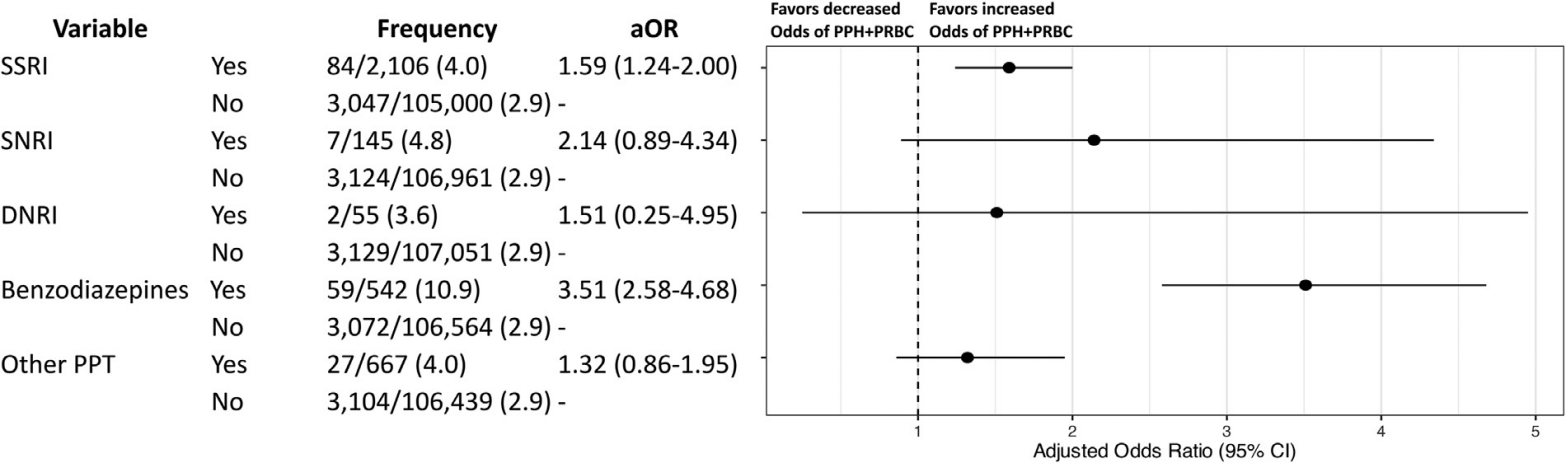
Regression model evaluating relationship between individual PPT drug classes and their association with postpartum hemorrhage requiring packed red blood cells (PPH+pRBC) *The following variables were adjusted for in the model, but the results are not shown: AWHONN PPH risk factors on admission, advanced maternal age, obesity, public health insurance, and race and ethnicity group.

When individual PPT drug classes were analyzed, monotherapy with SSRIs and benzodiazepines, but not SNRIs or DNRIs, were associated with PPH+pRBC (Figure 4). Among these drug classes, treatment with benzodiazepine monotherapy had the highest odds of PPH+pRBC (aOR 3.51, 95% CI: 2.58–4.68), occurring in 10.9% of exposed pregnancies (n=59/542).

Jackson. Association between psychopharmacotherapy and postpartum hemorrhage. Am J Obstet Gynecol MFM 2024.

**Figure 5:**
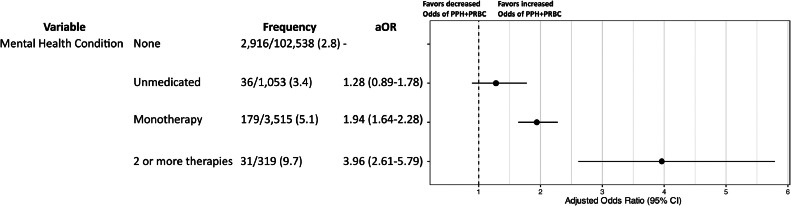
Regression model evaluating the relationship between treatment intensity of mental health condition and postpartum hemorrhage requiring packed red blood cells (PPH+pRBC) *The following variables were adjusted for in the model, but the results are not shown: AWHONN PPH risk factors on admission, advanced maternal age, obesity, public health insurance, and race and ethnicity group.

Patients receiving combination PPT experienced PPH+pRBC more frequently than those receiving monotherapy (9.7% vs. 5.1%; Figure 5). Compared to patients without a mental health condition, monotherapy was associated with nearly 2-fold increased odds and combination PPT was associated with nearly 4-fold greater odds of PPH+pRBC after adjustment for confounding variables (monotherapy: aOR 1.94, 95% CI: 1.64–2.28; combination PPT: aOR 3.96, 95% CI: 2.61–5.79). In this model, patients with untreated mental health conditions (no PTT) had no increased odds of PPH+pRBC compared to those without mental health conditions.

Jackson. Association between psychopharmacotherapy and postpartum hemorrhage. Am J Obstet Gynecol MFM 2024.

In the model evaluating a more broadly defined secondary outcome, PPT was positively associated with PPH+intervention after adjusting for covariates (aOR 1.53, 95% CI: 1.35–1.73; Supplemental Table 1).

### Supplemental analysis

The “expanded model,” which adjusted for additional variables, again demonstrated a positive association between PPT and PPH+pRBC (Supplemental Table 2). Among patients without any PPT exposure, mental health condition was not associated with PPH+pRBC after adjusting for covariates (Supplemental Table 3).

## Comment

### Principal findings

After systematically adjusting for hemorrhage risk factors at the time of admission for delivery, we observed that prenatal exposure to PPT is positively associated with PPH+pRBC. When individual PPT drug classes were evaluated, SSRIs and benzodiazepines were associated with PPH+pRBC, but DNRIs and SNRIs were not. Benzodiazepine monotherapy was associated with higher odds of PPH+pRBC than SSRI monotherapy and 3-fold increased odds compared to patients without PPT exposure. Combination PPT was associated with greater odds of PPH+pRBC than monotherapy and approximately 4-fold increased odds compared to patients without a mental health condition. Among patients without any prenatal exposure to PPT, the odds of PPH+pRBC did not differ between those with and without mental health conditions.

### Results in the context of what is known

The association between SSRIs, the most commonly used class of antidepressant medication in pregnancy, and PPH has been the subject of several investigations.^14^, ^15^, ^16^, ^17^, ^18^, ^19^, ^20^ Hanley et al. conducted a population-based cohort study of pregnancies in British Columbia, Canada, between 2002 and 2011, and concluded that SSRI use in the final month of pregnancy was not associated with PPH.^17^ Skalkidou et al. performed a national register-based cohort study in Sweden and concluded that patients who had any SSRI exposure during pregnancy, in any trimester, were at 1.33-fold increased odds of PPH.^14^ A systematic review and meta-analysis by Jiang et al., which included the above studies, similarly concluded that there was a 1.32-fold higher odds of PPH after pooling estimates but noted that there was considerable heterogeneity across the 8 included studies.^25^ Thus, existing evidence remains inconclusive.

Comparisons between studies are often hindered by study design factors. First, the definition of PPH used in most prior studies, blood loss of more than 500 mL for vaginal delivery or more than 1000 mL for cesarean delivery, is based on subjective, visual estimates of blood loss^14^^,^^18^ or PPH diagnosis codes that reflect that definition.^17^ These cutoffs, long used in clinical practice, do not imply that further resources or personnel were necessary for management. By using more rigorous and clinically relevant outcome definitions that restrict PPH cases to the subset that required blood transfusion or multiple uterotonic medications, we increase the reliability and validity of our findings. Second, previous studies do not adequately adjust for confounding factors, often omitting standard variables used for PPH risk stratification. In our analysis, we adjust for variables included in the AWHONN risk assessment tool, which is widely implemented nationally and endorsed by the Safe Motherhood Initiative of American College of Obstetricians and Gynecologists (ACOG) District II.^26^^,^^27^ Third, SSRI exposure is variably defined, sometimes including any reported use during pregnancy (any trimester), and elsewhere only including late-pregnancy exposure. Fourth, the individual SSRI drug prevalence varies substantially between study populations and there may be different effects on PPH risk between individual medications within a drug class. In Hanley et al., citalopram was the most frequently used SSRI, constituting 30.7% of SSRI use,^17^ whereas in our population, sertraline was the most frequently used medication, representing 53.8% of SSRI use.

Based on our review of the literature, no prior studies have investigated whether benzodiazepine exposure is associated with PPH.^28^^,^^29^ The pathophysiologic mechanisms by which benzodiazepines may contribute to bleeding are therefore poorly understood. Prior studies have noted that some benzodiazepines inhibit platelet activation and some cause vasodilation-induced hypotension.^30^^,^^31^ Benzodiazepines are agonists to the GABA receptor, which is present in uterine myometrium.^32^ Other GABA agonists have been found to inhibit uterine contractions in animal models.^33^ It is possible this action would contribute to uterine atony and subsequent hemorrhage. Nevertheless, other mechanisms may affect uterine tone or coagulation pathways.

### Clinical implications

Our findings have immediate implications for clinical practice by better facilitating patient education regarding risks of PPT during pregnancy and enabling patients to make more informed decisions about their plan of care. Prenatal use of SSRIs, benzodiazepines, or combination PPT should be incorporated into PPH risk assessment tools, as their strength of the association is larger than many other conditions included in those models.

Future prospective observational studies should seek to confirm our findings across diverse populations and settings. To definitively establish causality and demonstrate that prenatal PPT exposure causes PPH+pRBC, randomized controlled trials would be necessary; however, ethically designing such studies, where psychiatric treatment is withheld, would not be feasible. The underlying mechanisms by which such medications affect bleeding must be better elucidated, particularly for benzodiazepine therapy. Subgroup analysis should be performed to analyze outcomes associated with individual medications within each PPT class. There may be substantial differences in PPH risk between medications that are considered therapeutically similar. Intervention and management strategies for patients with prenatal PPT exposure should be explored to reduce PPH risk.

### Strength and limitations

This study has limitations, including its retrospective design and partial use of administrative data. Medication dosage, duration, and adherence were not evaluated but proximity to delivery is implicit by inclusion on the medication reconciliation completed at the time of hospitalization. Plasma drug concentrations and potential interactions with other medications were similarly not evaluated. Also, due to stigma surrounding mental health conditions, such as possible fear of repercussions for use of benzodiazepines, or off label use, it is possible there was underreporting of PPT. This limitation made it difficult to identify patients with mental health conditions who did not require PPT, however in a subanalysis excluding patients with PPT exposure we did not note any increased likelihood of PPH. Additionally, it is unclear why many patients were exposed to benzodiazepine monotherapy rather than concurrent therapy with another class such as SSRI, SNRI, and DNRI. It is possible that some patients on benzodiazepine monotherapy discontinued an SSRI, SNRI, or DNRI during the pregnancy. For patients on combination therapy, it was not possible to determine which specific combinations carried a higher risk for postpartum hemorrhage due to the large number of possible combinations. This study's focus on evaluating PPH+pRBC, rather than PPH alone, may limit direct comparability with some previous research. However, the inclusion of PPH+intervention as a secondary outcome helps address this limitation. Moreover, this approach strengthens our results by improving our ability to detect more meaningful associations that require clinical management.

### Conclusions

In this retrospective cross-sectional study, we observed an increased odds of clinically significant PPH associated with PPT exposure in late pregnancy. Monotherapy with either an SSRI or benzodiazepine medication is associated with PPH+pRBC. Combination PPT is associated with higher odds of PPH+pRBC than monotherapy. Our findings should stimulate further risk mitigation strategies but should not deter effective treatment of perinatal depression as lack of treatment is associated with adverse maternal and neonatal outcomes.^34^

## Consent statement

The local institutional review board approved this study as minimal-risk research and waived the requirement for informed consent.

## CRediT authorship contribution statement

**Frank I. Jackson:** Conceptualization, Data curation, Formal analysis, Investigation, Methodology, Visualization, Writing – original draft. **Insaf Kouba:** Conceptualization, Methodology, Writing – review & editing. **Natalie Meirowitz:** Conceptualization, Supervision, Writing – review & editing. **Nathan A. Keller:** Methodology, Writing – review & editing. **Luis A. Bracero:** Conceptualization, Supervision, Writing – review & editing. **Matthew J. Blitz:** Conceptualization, Data curation, Formal analysis, Investigation, Methodology, Project administration, Supervision, Writing – original draft.

## Appendix Supplementary materials

Image, application 1

## Footnotes

Supplementary material associated with this article can be found in the online version at doi:10.1016/j.xagr.2024.100402.
