# Supplementary material for: Association between psychopharmacotherapy and postpartum hemorrhage
Source: AJOG Glob Rep. 2024 Oct 9;4(4):100402. doi: 10.1016/j.xagr.2024.100402 (PMC11541838; doi:10.1016/j.xagr.2024.100402)
Supplement: Supplementary file 1 [file mmc1.docx]

**Supplemental Table 1**. Association between psychopharmacotherapy (PPT) and postpartum hemorrhage requiring intervention (PPH+intervention), which includes transfusion of packed red blood cells and/or use of two uterotonic agents in addition to routine postpartum oxytocin.

| **Characteristic** | **PPH+Intervention** | **Adjusted Odds Ratio (95% CI)** |
| --- | --- | --- |
| Psychopharmacotherapy |  |  |
| Yes | 321/3834 (8.4) | 1.53 (1.35-1.73) |
| No | 6202/103571 (6) | - |
| Induction |  |  |
| Yes | 2433/37187 (6.5) | 1.29 (1.22-1.36) |
| No | 4090/70218 (5.8) | - |
| Multiple gestation |  |  |
| Yes | 318/1965 (16.2) | 2.91 (2.56-3.3) |
| No | 6205/105440 (5.9) | - |
| ≥4 vaginal deliveries |  |  |
| Yes | 214/3250 (6.6) | 1.2 (1.03-1.39) |
| No | 6309/104155 (6.1) | - |
| Prior uterine surgery |  |  |
| Yes | 1261/17028 (7.4) | 1.18 (1.1-1.27) |
| No | 5262/90377 (5.8) | - |
| Large fibroids |  |  |
| Yes | 144/1346 (10.7) | 1.62 (1.34-1.94) |
| No | 6379/106059 (6) | - |
| Polyhydramnios |  |  |
| Yes | 144/1496 (9.6) | 1.56 (1.3-1.87) |
| No | 6379/105909 (6) | - |
| Fetal demise |  |  |
| Yes | 49/329 (14.9) | 1.76 (1.25-2.43) |
| No | 6474/107076 (6) | - |
| Anticoagulation |  |  |
| Yes | 59/656 (9) | 1.37 (1.03-1.79) |
| No | 6464/106749 (6.1) | - |
| Previa/Accreta/Abruption |  |  |
| Yes | 445/2121 (21) | 3.97 (3.55-4.44) |
| No | 6078/105284 (5.8) | - |
| Anemia |  |  |
| Yes | 847/6274 (13.5) | 2.42 (2.23-2.62) |
| No | 5676/101131 (5.6) | - |
| Thrombocytopenia |  |  |
| Yes | 96/502 (19.1) | 3.72 (2.93-4.67) |
| No | 6427/106903 (6) | - |
| AMA |  |  |
| Yes | 2214/33063 (6.7) | 1.1 (1.04-1.17) |
| No | 4309/74342 (5.8) | - |
| Obesity |  |  |
| Yes | 3502/51042 (6.9) | 1.19 (1.13-1.25) |
| No | 3021/56363 (5.4) | - |
| Public insurance |  |  |
| Yes | 2452/35455 (6.9) | 1.07 (1.01-1.14) |
| No | 3831/66425 (5.8) | - |
| Race and ethnicity |  |  |
| Non-Hispanic White | 2139/46597 (4.6) | - |
| Non-Hispanic Black | 980/13166 (7.4) | 1.47 (1.35-1.6) |
| Hispanic | 1572/20115 (7.8) | 1.69 (1.57-1.82) |
| Asian and Pacific Islander | 977/14168 (6.9) | 1.6 (1.47-1.74) |
| Native American and Alaska Native | 34/638 (5.3) | 1.13 (0.77-1.6) |
| Multiracial or Other | 603/9174 (6.6) | 1.47 (1.33-1.62) |
| Unknown or declined | 218/3547 (6.1) | 1.3 (1.11-1.5) |

Data are number cases/group (percent) unless otherwise specified

**Supplemental Table 2**. Expanded model: association between psychopharmacotherapy (PPT) and postpartum hemorrhage requiring packed red blood cells (PPH+pRBC) with additional adjustment for chorioamnionitis, mode of delivery, macrosomia, hypertensive disorders of pregnancy, and excessive gestational weight gain.

| **Characteristic** | **PPH+pRBC** | **Adjusted Odds Ratio (95% CI)** |
| --- | --- | --- |
| Psychopharmacotherapy |  |  |
| Yes | 321/3834 (8.4) | 1.48 (1.29-1.69) |
| No | 6202/103571 (6) | - |
| Induction |  |  |
| Yes | 2433/37187 (6.5) | 1.33 (1.25-1.42) |
| No | 4090/70218 (5.8) | - |
| Multiple gestation |  |  |
| Yes | 318/1965 (16.2) | 2.53 (2.2-2.9) |
| No | 6205/105440 (5.9) | - |
| ≥4 vaginal deliveries |  |  |
| Yes | 214/3250 (6.6) | 1.38 (1.17-1.62) |
| No | 6309/104155 (6.1) | - |
| Prior uterine surgery |  |  |
| Yes | 1261/17028 (7.4) | 0.95 (0.87-1.03) |
| No | 5262/90377 (5.8) | - |
| Large fibroids |  |  |
| Yes | 144/1346 (10.7) | 1.61 (1.33-1.94) |
| No | 6379/106059 (6) | - |
| Polyhydramnios |  |  |
| Yes | 144/1496 (9.6) | 1.37 (1.13-1.65) |
| No | 6379/105909 (6) | - |
| Fetal demise |  |  |
| Yes | 49/329 (14.9) | 2.12 (1.48-2.98) |
| No | 6474/107076 (6) | - |
| Anticoagulation |  |  |
| Yes | 59/656 (9) | 1.3 (0.95-1.74) |
| No | 6464/106749 (6.1) | - |
| Previa/Accreta/Abruption |  |  |
| Yes | 445/2121 (21) | 3.49 (3.09-3.93) |
| No | 6078/105284 (5.8) | - |
| Anemia |  |  |
| Yes | 847/6274 (13.5) | 2.22 (2.03-2.42) |
| No | 5676/101131 (5.6) | - |
| Thrombocytopenia |  |  |
| Yes | 96/502 (19.1) | 3.23 (2.5-4.14) |
| No | 6427/106903 (6) | - |
| AMA |  |  |
| Yes | 2214/33063 (6.7) | 1.06 (1-1.13) |
| No | 4309/74342 (5.8) | - |
| Obesity |  |  |
| Yes | 3502/51042 (6.9) | 1.04 (0.98-1.11) |
| No | 3021/56363 (5.4) | - |
| Excessive gestational weight gain |  |  |
| Yes | 3122/47293 (6.6) | 1.07 (1.01-1.14) |
| No | 2690/48108 (5.6) | - |
| Public Insurance |  |  |
| Yes | 2452/35455 (6.9) | 1.1 (1.03-1.17) |
| No | 3831/66425 (5.8) | - |
| Race and ethnicity group |  |  |
| Non-Hispanic White | 2139/46597 (4.6) | - |
| Non-Hispanic Black | 980/13166 (7.4) | 1.46 (1.33-1.6) |
| Hispanic | 1572/20115 (7.8) | 1.71 (1.57-1.85) |
| Asian and Pacific Islander | 977/14168 (6.9) | 1.61 (1.47-1.75) |
| Native American and Alaska Native | 34/638 (5.3) | 1.24 (0.84-1.77) |
| Multiracial or Other | 603/9174 (6.6) | 1.45 (1.31-1.61) |
| Unknown or declined | 218/3547 (6.1) | 1.22 (1.03-1.43) |
| Cesarean Delivery |  |  |
| Yes | 3243/36576 (8.9) | 1.7 (1.59-1.81) |
| No | 3268/70560 (4.6) | - |
| Chorioamnionitis |  |  |
| Yes | 253/1735 (14.6) | 2.58 (2.22-2.99) |
| No | 6270/105670 (5.9) | - |
| Pre-eclampsia with severe features |  |  |
| Yes | 572/4717 (12.1) | 1.53 (1.37-1.69) |
| No | 5951/102688 (5.8) | - |
| Other hypertensive disorder |  |  |
| Yes | 827/13750 (6) | 0.88 (0.81-0.96) |
| No | 5696/93655 (6.1) | - |
| Macrosomia |  |  |
| Yes | 670/6849 (9.8) | 1.34 (1.15-1.55) |
| No | 5853/100556 (5.8) | - |

Data are number cases/group (percent) unless otherwise specified

*This model should be interpreted cautiously because some of the additional variables are mediators between exposure and outcome.

*Macrosomia defined as birthweight >4000 grams.

*Excessive gestational weight gain defined as weight gain exceeding the upper limit recommended by the Institute of Medicine based on pre-pregnancy body mass index.

**Supplemental Table 3**. Association of mental health condition, in the absence of PPT exposure, with postpartum hemorrhage requiring packed red blood cells (PPH+pRBC), adjusting for covariate factors.

| **Characteristic** | **PPH+pRBC** | **Adjusted Odds Ratio (95% CI)** |
| --- | --- | --- |
| Mental health condition |  |  |
| Yes | 36/1053 (3.4) | 1.28 (0.89-1.78) |
| No | 2914/102518 (2.8) | - |
| Induction |  |  |
| Yes | 968/35865 (2.7) | 1.19 (1.09-1.3) |
| No | 1982/67706 (2.9) | - |
| Multiple gestation |  |  |
| Yes | 185/1886 (9.8) | 3.37 (2.85-3.96) |
| No | 2765/101685 (2.7) | - |
| ≥4 vaginal deliveries |  |  |
| Yes | 76/3109 (2.4) | 0.94 (0.73-1.18) |
| No | 2874/100462 (2.9) | - |
| Prior uterine surgery |  |  |
| Yes | 741/16385 (4.5) | 1.43 (1.3-1.58) |
| No | 2209/87186 (2.5) | - |
| Large fibroids |  |  |
| Yes | 94/1303 (7.2) | 2.27 (1.8-2.82) |
| No | 2856/102268 (2.8) | - |
| Polyhydramnios |  |  |
| Yes | 76/1394 (5.5) | 1.9 (1.48-2.4) |
| No | 2874/102177 (2.8) | - |
| Fetal demise |  |  |
| Yes | 21/279 (7.5) | 1.64 (0.99-2.59) |
| No | 2929/103292 (2.8) | - |
| Anticoagulation |  |  |
| Yes | 32/606 (5.3) | 1.52 (1.02-2.18) |
| No | 2918/102965 (2.8) | - |
| Previa/Accreta/Abruption |  |  |
| Yes | 289/2013 (14.4) | 5.56 (4.84-6.37) |
| No | 2661/101558 (2.6) | - |
| Anemia |  |  |
| Yes | 640/5988 (10.7) | 4.27 (3.87-4.7) |
| No | 2310/97583 (2.4) | - |
| Thrombocytopenia |  |  |
| Yes | 65/481 (13.5) | 5.39 (4.04-7.09) |
| No | 2885/103090 (2.8) | - |
| AMA |  |  |
| Yes | 1056/31575 (3.3) | 1.18 (1.08-1.28) |
| No | 1894/71996 (2.6) | - |
| Obesity |  |  |
| Yes | 1609/49159 (3.3) | 1.17 (1.09-1.27) |
| No | 1341/54412 (2.5) | - |
| Public insurance |  |  |
| Yes | 1233/34491 (3.6) | 1.29 (1.19-1.4) |
| No | 1626/63771 (2.5) | - |
| Race and ethnicity |  |  |
| Non-Hispanic White | 834/43930 (1.9) | - |
| Non-Hispanic Black | 577/12916 (4.5) | 1.89 (1.69-2.13) |
| Hispanic | 688/19740 (3.5) | 1.59 (1.42-1.78) |
| Asian and Pacific Islander | 431/13983 (3.1) | 1.63 (1.44-1.84) |
| Native American and Alaska Native | 21/630 (3.3) | 1.69 (1.03-2.6) |
| Multiracial or Other | 298/8949 (3.3) | 1.66 (1.44-1.91) |
| Unknown or declined | 101/3423 (3) | 1.42 (1.13-1.75) |

Data are number cases/group (percent) unless otherwise specified
